# Single-dose methotrexate for the treatment of ectopic pregnancy: Our experience from 2010 to 2015

**DOI:** 10.12669/pjms.331.11238

**Published:** 2017

**Authors:** Emre Erdem Tas, Gulin Feykan Yegin Akcay, Ayse Filiz Avsar

**Affiliations:** 1 Dr. Emre Erdem Tas, Department of Gynecology and Obstetrics, Yildirim Beyazit University, Ankara, Turkey; 2 Dr. Gulin Feykan Yegin Akcay, Department of Gynecology and Obstetrics, Ataturk Education and Research Hospital, Ankara, Turkey; 3 Prof. Dr. Ayse Filiz Avsar, Department of Gynecology and Obstetrics, Yildirim Beyazit University, Ankara, Turkey

**Keywords:** Ectopic pregnancy, β–human chorionic gonadotropin, Methotrexate, Single injection

## Abstract

**Objective:**

To evaluate the success of systemic single-dose methotrexate (MTX) treatment in patients with ectopic pregnancy (EP) and to investigate factors related to treatment success.

**Methods:**

This retrospective study had been performed in Yildirim Beyazit University between January 2010 and December 2015. Demographic and clinical characteristics, ultrasonografic findings, pretreatment serum β–human chorionic gonadotropin (β-hCG) and progesterone levels of 58 patients with EP were retrieved from hospital records retrospectively. The patients were grouped according to MTX treatment success (response *vs.* failure).

**Results:**

Single-dose MTX-treatment was successful in 72.4% (42/58) of patients. The mean pretreatment β-hCG level was significantly lower in responders than in failures (2080 ± 2322 *vs*. 5707 ± 3885 IU/L, *p* = 0.001), and 2678 IU/L was the most suitable cutoff to predict success (75% sensitivity, 73.8% specificity). Moreover, failure rate was 8.45 times more in group of patients whose β-hCG values were determined above the cutoff. The presence of fetal cardiac activity adversely affected treatment success (odds ratio = 12, *p* = 0.004). Treatment success was not affected by past history of ectopic pregnancy, thickness of endometrium, progesterone value or presences of pseudosac and free pelvic fluid.

**Conclusion:**

The success rate of single dose MTX in this study was 72.8 %, and we found that failure rate of MTX treatment was 8.45 times more in group of patients whose initial serum β-hCG values were above 2678 IU/L and 12 times more in patients with fetal cardiac activity

## INTRODUCTION

Blastocyst implantation outside the uterine cavity is defined as ectopic pregnancy (EP). According to the Centers for Disease Control and Prevention, 2% of all pregnancies in the US were ectopic.^1^ With the development of treatment options, the rate of maternal mortality due to EP declined by 57% from the 1980s to the 2000s; EP, however, remains the leading cause of first-trimester maternal mortality.^2^

EP can be treated surgically or non-surgically. Although surgical approaches are the mainstay of treatment, advances in early diagnosis have facilitated the introduction of methotrexate (MTX) therapy. In selected patients, MTX therapy is cost effective and achieves similar results with multi-dose MTX therapy in terms of success rate and future fertility.^3^ Moreover, drug-based therapy avoids the inherent risks of anesthesia and surgery.^4^ Success rates of MTX treatment for EP range from 76% to 100%.^5^–^8^ Preliminary reports have cited several factors potentially associated with MTX treatment failure, notably pretreatment β–human chorionic gonadotropin (β-hCG) level and the presence of fetal cardiac activity.^4^–^6^ The rationale of this study is to report our experience with single dose MTX use in patients with EP and to investigate pretreatment Bhcg, preogesterone levels and precense of fetal cardiac activity as there is a limited data especially about the relationship between these factors and treatment success’.

## METHODS

Data from 98 patients diagnosed with EP between January 2010 and December 2015 in Yildirim Beyazit University, who were treated by systemic MTX or surgery were reviewed retrospectively. The study protocol was approved by the Ethics Committee of our university. EP was diagnosed by inappropriately rising serum β-hCG level (slower doubling time on consecutive measurements) and adnexal mass or extrauterine tubal gestational sac without intrauterine gestation, as observed by transvaginal ultrasonography (TVU). Surgery was the first treatment option in patients with excessive free pelvic fluid (determined by TVU) or hemodynamic instability with signs and symptoms of acute abdomen. The remaining patients were treated with systemic single-dose MTX (50 mg/m^2^, i.m.); no patient was excluded from MTX therapy due to baseline serum β-hCG level or ultrasonic detection of fetal cardiac activity. Serum β-hCG concentrations were measured on days four and seven post-injection and weekly thereafter until a decline ≥ 15% and a concentration < 15 IU/L, indicating treatment success, were achieved.

Data on patients’ demographic and clinical characteristics (age, gravidity, parity, EP history, presenting symptoms and signs), TVU findings (endometrial thickness, pseudosac presence, fetal cardiac activity, free pelvic fluid), and pretreatment serum β-hCG and progesterone levels were retrieved from hospital records. The patients were grouped according to MTX treatment success (response *vs*. failure).

Statistical analyses were performed with SPSS version 20 (IBM Corp, Los Angeles, California, USA). The Kolmogorov–Smirnov test was used to assess the normality of data. Normally distributed data were expressed as mean ± standard deviation (range) and non-parametric data were expressed as median (interquartile range) (range). The independent-samples *t* test and Mann–Whitney *U* test were used to compare parametric and non-parametric data, respectively, between groups. Categorical variables were compared using the chi-squared test. Binary logistic regression analyses were used to assess influencing factors on treatment success. The significance level was *p* < 0.05, and odds ratios (ORs) were determined with 95% confidence intervals.

## RESULTS

**Fig. 1:**
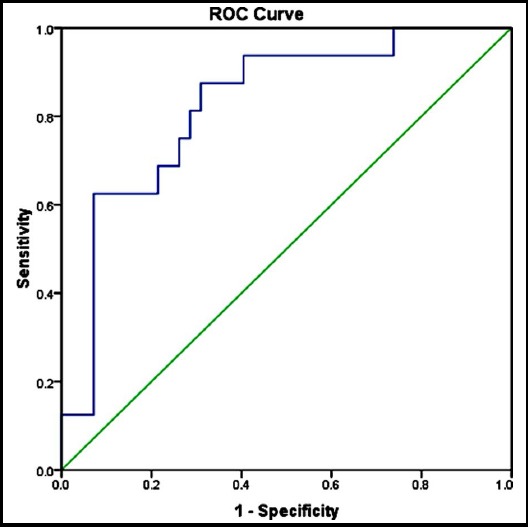
Receiver operating characteristic (ROC) curve for the ability of pretreatment β–human chorionic gonadotropin level to predict single-dose methotrexate treatment success (Area under curve = 0.82, standard error = 0.6, p < 0.001).

Systemic single-dose MTX was administered to 58 of 98 (59.2%) patients with EP, and was successful in 42 (72.4%) patients. Three (3%) patients in the treatment failure group received second MTX doses and 13 (13.3%) underwent surgery. Surgery was thus performed in 53 (54.1%) patients. Pretreatment β-hCG level differed significantly between the treatment response and failure groups (2080 *vs*. 5707 mIU/dL; *p* = 0.001). β-hCG value of 2678 IU/L was the most suitable cutoff for the prediction of treatment success (75% sensitivity, 73.8% specificity). Only the presence of fetal cardiac activity adversely affected treatment success, which was achieved in 25% (2/8) of patients showing such activity (OR = 12, *p* = 0.004).(Fig.1)

The characteristics of patients treated with single-dose MTX, overall and according to treatment success are summarizes in Table-I.

**Table-I:** Characteristics of patients treated with single-dose methotrexate, overall and according to treatment success.

Characteristic	Single-dose MTX (n = 58)	Treatment success (n = 42)	Treatment failure^α^ (n = 16)	p^β^	ORs
***Demographical Properties***					
Age (years)	30.9 ± 4.9 (21–44)	31.5 ± 5.2 (21–44)	29.2 ± 3.9 (22–39)	0.13	0.884
Gravidity	3 (2) (1–8)	3 (3) (1–8)	3 (2) (1–5)	0.28	1.18
Parity	1 (2) (0–3)	1 (2) (0–2)	2 (2) (0–3)	0.15	2.6
Previous ectopic pregnancy	9 (15.5%)	8 (19%)	1 (6.3%)	0.42	0.94
Gestational age (weeks)	6 (2) (3–12)	6 (2) (3–12)	6 (1) (3–8)	0.5	1.64
***Pretreatment Signs and symptoms***					
None	6 (10.3%)	3 (5.2%)	3 (5.2%)	0.33	0.6
Pelvic pain with/without vaginal bleeding	52 (89.7%)	39 (67.2%)	13 (22.5%)		
Pelvic pain only	20 (34.5%)	16 (27.6%)	4 (6.9%)		
Vaginal bleeding only	17 (29.3%)	12 (20.7%)	5 (8.6%)		
Pelvic pain with vaginal bleeding	15 (25.9%)	11 (19%)	4 (6.9%)		
***Laboratuvary Values and TVU Findings***					
β-hCG level (IU/L)	3080 ± 3244 (151–16,200)	2080 ± 2322 (151–10,360)	5707 ± 3885 (676–16,200)	0.001	8.45
Progesterone level (mg/dL)	4.45 (5.35) (0.98–21.1)	6 (7.1) (0.9–14.3)	4.2 (3.6) (1.2–21.1)	0.22	0.99
Endometrial thickness (mm)	7 (2) (3–16)	7 (2) (3–13)	7.5 (5.5) (4–16)	0.63	1.52
Pseudosac	9 (15.5%)	7 (16.7%)	2 (12.5%)	0.52	0.71
Fetal cardiac activity	8 (13.8%)	2 (4.7%)	6 (37.5%)	0.004	12
Free pelvic fluid	25 (43.1%)	20 (47.6%)	5 (31.3%)	0.37	0.5

Data are presented as mean ± standard deviation (range), median (interquartile range) (range), or *n* (%).

MTX: methotrexate; β-hCG: β–human chorionic gonadotropin.

ORs: Odds Ratios TVU: Transvaginal ultrasound

α Successful treatment required multiple MTX doses or surgical intervention.

β Treatment success *vs*. failure.

## DISCUSSION

MTX is a folic-acid antagonist that inactivates *de novo* synthesis of cellular DNA.^9^ This anti-neoplastic, anti-metabolic drug has been used increasingly to treat EP since Tanaka et al.^10^ first reported on it in 1982. A standard treatment option for many patients. Single- and multi-dose systemic MTX protocols are used most commonly, with no difference in success rate.^3^,^11^ Given its simplicity and low complication rate, the single-dose protocol is used more widely throughout the world.^12^

In literature, a β-hCG level exceeding 5000–6000 IU/L and the presence of fetal cardiac activity were marked as relative contraindications to systemic MTX treatment by a group of study.^13^,^14^ In our clinic and many others, MTX treatment has been using in EP patients with detected fetal cardiac activity and high β-hCG levels. On the other hand, definitive contraindications for MTX treatment can be listed as hemodynamic instability, acute abdomen signs/symptoms, intrauterine pregnancy, breast feeding, MTX sensitivity, peptic ulcer disease, active pulmonary disease, evidence of immunodeficiency, hepatic-renal or hematologic dysfunction and all researchers agree on this issue.^7^,^9^

Reported success rates of MTX regimes vary, due to many factors. Pretreatment β-hCG value is one of the most popular factor among those. The success rate has been shown to decrease with increasing pretreatment β-hCG level.^6^,^15^ Many cutoff values were proposed with different sensitivities and specificities in previous reports, but consensus on a cutoff value predicting treatment success is lacking. For instance, in this report we found a cutoff value, 2678 IU/L, with 75% sensitivity and 73.8% specificity; however, in a report by Mirbolouk et al.^14^, reported a success rate of single-dose MTX treatment similar to that achieved in this study (77.1%), established a much lower β-hCG cutoff (1375 IU/L) with similar sensitivity and specificity. These differences may be due to choosing characteristics of patients or may be due to indeterminate factor which have not been established, yet.

The presence of fetal cardiac activity is another major adverse factor, as demonstrated in this study. Erdem et al.^7^ reported no treatment success in patients with detectable fetal cardiac activity. In contrast,. Lipscomb et al.^16^ reported an 87.5% success rate in this group although the pretreatment serum β-hCG levels were three fold higher than our results (87.5% vs 25%.) Zargar et al.^17^ reported cardiac activity were much more responsive to single dose treatment. Due to the wide range of success^7^,^14^,^16^, especially in patients with detected fetal cardiac activity, we suggest MTX treatment in clinics which have emergency operating conditions preferably with laparoscopy after obtaining informed consent from patients and preparations of blood components preoperatively. The success of systemic MTX treatment may decrease with increasing endometrial thickness, but the significance of this effect is controversial. Some authors have reported significant effects, attributing them to higher β-hCG levels reflected by thicker endometrium,^18^,^19^ whereas this study and others demonstrated no significant correlation (p=0.63, OR: 1.52).^14^ One previous study investigated the relation between pseudosac presence and systemic MTX treatment success, and authors found that pseudosacs increased the risk of treatment failure 4.24 times.^20^ Conversely, pseudosacs were more prevalent in our treatment response group, although this variable was not correlated with treatment success (*p*=0.52, OR=0.71). Age (which increases EP risk), reproductive profile and progesterone values do not affect MTX treatment success, as demonstrated in this and previous studies.^3^,^6^,^7^,^14^,^16^,^21^-^26^ Moreover, presence of pelvic pain with/without vaginal bleeding have no effect on treatment success (*p* = 0.33).

Many authors regard the presence of pelvic fluid (presumably blood with/without clotting) on ultrasound to contraindicate medical therapy because of concern that it may indicate ongoing rupture of EP. However, systemic MTX is safe in hemodynamically stable patients without excessive fluid or clotting, and fluid presence does not affect treatment success, as demonstrated in this study and others.^12^,^24^,^25^ The limitation regarding this study is that we could not determine amounts of pelvic fluid.

MTX is a promising agent for the treatment of EP, but surgery remains the main treatment option for many patients. More than half of patients with EP require surgery.^7^,^21^,^25^ Laoparoscopy is the most commonly advocated approach in these cases, but many clinics have not used it effectively.^20^,^22^,^25^ In our clinic, laparoscopy has been performed in 77.3% of patients with EP.

## CONCLUSION

The success rate of single dose MTX in this study was 72.8%, and we found that failure rate of MTX treatment was 8.45 times more in group of patients whose initial serum β-hCG values were above 2678 IU/L and 12 times more in patients with fetal cardiac activity. Indeed; especially in patients with fetal cardiac activity, we suggest MTX treatment in clinics, which have emergency operating conditions preferably with laparoscopy after obtaining informed consent from patients and preparations of blood components preoperatively.
